# Intensive Care Resources and 60-Day Survival of Critically-Ill COVID-19 Patients

**DOI:** 10.7759/cureus.13210

**Published:** 2021-02-07

**Authors:** Corinna N Lang, Viviane Zotzmann, Bonaventura Schmid, Michael Berchtold-Herz, Stefan Utzolino, Paul Biever, Daniel Duerschmied, Christoph Bode, Tobias Wengenmayer, Dawid L Staudacher

**Affiliations:** 1 Department of Cardiology and Angiology I (Heart Center Freiburg University - Bad Krozingen), Medical Center – University of Freiburg, Faculty of Medicine, Freiburg, DEU; 2 Department of Medicine III (Interdisciplinary Medical Intensive Care), Medical Center – University of Freiburg, Faculty of Medicine, Freiburg, DEU; 3 Department of Emergency Medicine, Medical Center – University of Freiburg, Faculty of Medicine, Freiburg, DEU; 4 Department of Cardiovascular Surgery (Heart Center Freiburg University – Bad Krozingen), Medical Center – University of Freiburg, Faculty of Medicine, Freiburg, DEU; 5 Department of General and Visceral Surgery, Medical Center – University of Freiburg, Faculty of Medicine, Freiburg, DEU

**Keywords:** covid-19, ards, 60-day survival, icu resources

## Abstract

Background: Germany reported sufficient intensive care unit (ICU) resources throughout the first wave of coronavirus disease 2019 (COVID-19). The treatment of critically ill COVID-19 patients without rationing may improve the outcome. We therefore analyzed ICU resources allocated to COVID-19 patients with respiratory failure and their outcomes.

Methods: Retrospectively, we enrolled severe acute respiratory syndrome coronavirus 2 (SARS-CoV-2) polymerase chain reaction (PCR)-positive patients with respiratory failure from 03/08/2020 to 04/08/2020 and followed until 05/28/2020 in the university hospital of Freiburg, Germany.

Results: In the defined interval, 34 COVID-19 patients were admitted to the ICU with median age of 67±13 (31-86) years. Six of 34 (17.6%) were female. All patients suffered from moderate or severe acute respiratory distress syndrome (ARDS), 91.2% of the patients were intubated and 23.5% required extracorporeal membrane oxygenation (ECMO). Proning was performed in 67.6%, renal replacement therapy (RRT) was required in 35.3%. Ninety-six percent required more than 20 nursing hours per day. Mean ICU stay was 21±19 (1-81) days. Sixty-day survival of critically ill COVID-19 patients was 50.0% (17/34). Causes of death were multi-organ failure (52.9%), refractory ARDS (17.6%) and intracerebral hemorrhage (17.6%).

Conclusions: Treatment of critically ill COVID-19 patients is protracted and resource-intense. In a context without resources shortage, 50% of COVID-19 with respiratory failure survived up to 60 days.

## Introduction

Severe acute respiratory coronavirus 2 (SARS-CoV-2) pandemic encountered southwestern Germany in March 2020, causing relevant case numbers of the coronavirus disease 2019 (COVID-19)-related pneumonia. Clinical courses of critically ill patients with COVID-19 were characterized in international cohorts with hospital mortality rates between 50 and 88% (including ongoing treatment cohorts) [1-3]. The overall case fatality rates vary internationally (1-12%). Recently, reported case fatality rates in Germany remain lower than in neighboring countries [3-5].

Patients with severe pulmonary failure and adult respiratory distress syndrome (ARDS) are reported to require multiple organ replacement therapies, some of which have to be applied at the same time. As recently reported, severity of COVID-19 cases immensely increases the strain on staff and resources [1].

This retrospective study analyzes the first cohort of patients on three intensive care units at a third-level treatment center in Freiburg, Germany. During the observation period the center never faced a shortage of human or technical resources for the treatment of those patients with ARDS caused by COVID-19. The present study focuses on the necessary resources for the treatment and 60-day survival. 

This article was previously posted to the Research Square preprint server 2020, August 02.

## Materials and methods

We enrolled critically ill patients in case of polymerase chain reaction (PCR)-confirmed SARS-CoV-2 infection and intensive care unit (ICU) admission due to pulmonary failure caused by COVID-19 from 03/08/2020 (date of ICU admission of our first case) to 04/08/2020.

Inclusion criteria

According to the local pandemic plan, COVID-19 patients were treated at different ICUs (medical and surgical). Patients were admitted to the ICU from the emergency room (ER), regular hospital wards, or from first and secondary treatment centers. Our center serves as a reference and referral center for ARDS and veno-venous extracorporeal membrance oxygenation (ECMO) offering a 24/7 ECMO retrieval service. All patients with proven SARS-CoV-2 infection and severe respiratory failure requiring ICU therapy were included in the present study. The PCR tests were carried out from nasopharyngeal swabs or tracheal secretion. Patients with high clinical probability of COVID-19 but negative virology testing were excluded.

Patients with do not resuscitate (DNR) orders not excluding ICU therapy for respiratory failure (ventilation, prone positioning) were included. We adhered to their wish if patients did prefer to not continue ICU therapy. 

Pre-ICU treatment

Patient management prior to ICU was outlined in in-house standard operating procedures related to COVID-19. It included hygiene concepts, laboratory work-up, lung-ultrasound, radiology imaging, volume therapy, perioperative management, discharge requirements, and anti-infective recommendations. During the first wave of the pandemic all guidelines were rapidly implemented and shared with transferring hospitals to align therapies.

If patients were transferred from other hospitals, we collected patients reports to minimize the loss of information.

ARDS treatment

The ARDS treatment followed current guidelines and house-intern standard operating procedures for lung-protective ventilation, prone positioning and supportive therapies [6,7].

In our center attempts with non-invasive ventilation were carried out with high-flow nasal cannula (HFNC) or non-invasive ventilation (NIV) prior to invasive mechanical ventilation (IMV).

At this point of time, steroids next to relaxation or recruitment maneuvers were not standard therapy in ARDS in our center; these strategies remained a case-by-case decision and were rarely used in early ARDS. Steroid therapies have been implemented later on.

In case of persistent hypoxia or hypercapnia in patients with lung-protective ventilation after employment of all conservative strategies, ECMO was evaluated by an interdisciplinary team of at least one ECMO specialist, a registered nurse, and a perfusionist following local standards. (Relative) exclusion criteria were ventilation over seven days without lung protection, acute intracerebral hemorrhage, severe comorbidities (metastatic cancer), severe thrombocytopenia, coagulopathy, and advanced age (no cut-off).

For the ECMO circuit we cannulated bifemoral or jugular with a dual lumen cannula. Applied systems were Stöckert Centrifugal Pump Console (SCPC) (LivaNova, Munich, Germany) or the Cardiohelp-System (Maquet, Rastatt, Germany) with customized tubing sets and oxygenators. We primed the sets with 700 ml electrolyte solution and 5000 IE of unfractionated heparin.

Nursing hours

Nursing hours were calculated on a representative day for the patients and cumulative for one representative medical ICU (14-bed) based on the Inpuls® Intensivpflege- und Leistungserfassungssystem, intensive care nursing and activity recording system (Inpuls®, Heidelberg, Germany). The cumulative calculation comprises day by day categorization of the patients in six defined categories (K1-6) related to defined criteria. Each category results in ascending requirements for nursing hours.

Category 1 comprises patients with monitoring indication and without complications (nursing hours 7.33 hours in 24 hours). Patients with monitoring indication and with complications are grouped in category 2 (8.40 nursing hours in 24 hours). Categories 3-6 capture patients with organ replacement therapies. Category 3 depicts a patient with e.g. only short period of invasive ventilation (perioperative etc.) with 11.00 nursing hours in 24 hours. A patient with a permanent organ replacement (ventilation or renal replacement therapy (RRT)) and e.g. large wound areas is categorized in the 4th category (13.85 nursing hours in 24 hours). Seriously ill patients with IMV and circulatory support systems, therapeutic positioning maneuvers, permanent medication/transfusion requirements belong to the 5th category (20.25 nursing hours in 24 hours). Highly critical patients with permanent two or more organ replacement therapies, permanent medication requirements and e.g. assistance in diagnostic or therapeutic interventions reach category 6 (21.66 nursing hours in 24 hours).

The criteria for defining the categories are assigned to subgroups (consciousness, breathing, hygiene, positioning, medication, wounds etc.). Critical events result in upgrading (cardio-pulmonal resuscitation, massive hemorrhage, pericardial effusion etc.). Lone or combined organ replacement categories result in certain categories.

Endpoint definition

ARDS was classified according to the Berlin classification [8]. Acute kidney injury was diagnosed according to the RIFLE definition and supported by RRT if necessary [9,10]. Frailty was clinically judged according to the modified Rockwood Clinical Frailty Score if meeting the 5th category or higher [11]. All patients dismissed home or to a rehabilitation unit were considered 60-day survivors in regards to this study (if ICU and following hospital stay were shorter than 60 days). The cause of death was determined by clinical judgment of at least two intensivists (participants of this research project).

Data management

The data collection was carried out till 05/28/2020 for the patients admitted between 08/03/2020 until 08/04/2020. Patients admitted later were not included in the analysis. We extracted patient related data from our hospital data systems and documentation from transferring hospitals. The local ethics committee approved the study protocol (Ethik-Kommission der Albert-Ludwigs-Universität Freiburg file number 234/20). Non-survivors and ICU-survivors were compared with chi-square test, Student´s t-test, and Fisher´s exact. We performed analyses with Statistical Package for Social Sciences (SPSS) version 26 (IBM Corp., Armonk, NY, USA) and Prism version 8 (GraphPad, San Diego, CA, USA) and a p-value of <0.05 was considered statistically significant.

## Results

Baseline characteristics and comorbidities

During the observation period, 34 COVID-19 patients with pulmonary failure were admitted and treated on the ICU. Medium age was 67±13 years, six (17.6%) were female (Table 1).

**Table 1:** Baseline Characteristics Abbreviations: HbA1c, hemoglobin A1c; ICU, intensive care unit; BMI, body mass index (calculated in kilograms divided by height in meters squared); ER, emergency room; SOFA score, Sequential Organ Failure Assessment Score; SAPS II score, Simplified Acute Physiology Score; TISS Score, Therapeutic Intervention Scoring System; Inpuls®, Intensivpflege- und Leistungserfassungssystem, intensive care nursing and activity recording system (Inpuls®, Heidelberg, Germany); ARDS, acute respiratory distress syndrome in accordance with the Berlin classification [8]. Baseline characteristics are displayed for all patients, non-survivors and survivors. Data are n (%) or mean with standard deviation and range. Student´s t-test or Fisher´s exact test was performed to derive p-values. a DNR, no not resuscitate order was assessed prior to ICU admission and did not exclude ICU therapy for respiratory failure (ventilation, prone positioning). Patients not willing to receive ICU therapy were primarily not transferred to our unit. b Other cardiac condition: valve operation or chronic heart failure. c Frailty was clinically judged; clinical frailty scale (CFS) which is a modification of the Canadian frailty scale by Rockwood [11]. If a category of 5 or higher was met (help needed for daily life activities), we categorized the patient as frail.

	all patients (n=34)	non-survivors (n=17)	survivors (n=17)	p-value
number of patients	34	17 (50%)	17 (50%)	
age, years	67±13 (31-86)	70±10 (49-86)	64±14 (31-84)	0.212
age range, years				
30-49	3 (8.8%)	1 (5.8%)	2 (11.8%)	
50-59	6 (17.6%)	2 (11.8%)	4 (23.5%)	
60-69	9 (26.5%)	5 (29.4%)	4 (23.5%)	
70-79	11 (32.4%)	6 (35.3%)	5 (29.4%)	
≥80	5 (14.7%)	3 (17.6%)	2 (11.8%)	
sex				
female	6 (17.6%)	4 (23.5%)	2 (11.8%)	0.656
health insurance				
mandatory	25 (73.5%)	13 (76.4%)	12 (70.6%)	0.697
private	9 (26.5%)	4 (23.6%)	5 (39.4%)	0.697
DNR order^a^	5 (14.7%)	4 (23.5%)	1 (5.9%)	0.335
location of admisson				
home/ER	5 (14.7%)	4 (23.5%)	1 (5.9%)	0.335
internal hospital ward	15 (44.1%)	6 (35.3%)	9 (52.9%)	0.491
hospital transfer	14 (41.2%)	7 (41.2%)	7 (41.2%)	1.00
severity scores				
SOFA score	8.9±3.6 (2-16)	10.0±1.9 (6-12)	7.8±4.6 (2-16)	0.083
SAPS II score	46±12 (15-70)	50±7 (42-70)	42±14 (15-69)	0.037
TISS score	14.8±7 (0-31)	16±5 (10-28)	8±23 (0-31)	0.359
Inpuls®	5.3±0.7 (3-6)	5.5±0.5 (5-6)	5.2±0.8 (3-6)	0.217
ARDS				
mild	0	0	0	
moderate	17 (50%)	7 (41.2%)	10 (58.8%)	0.494
severe	17 (50%)	10 (58.8%)	7 (41.2%)	0.494
comorbidites				
BMI (kg/m²)	28±4.6 (18-39)	29±5.5 (18-39)	28±3.7 (23-35)	0.913
≥26 (kg/m²)	26 (76.5%)	13 (76.5%)	13 (76.5%)	1.00
≥30 (kg/m²)	11 (32.4%)	6 (35.3%)	5 (29.4%)	1.00
hypertension	18 (52.9%)	9 (52.9%)	9 (52.9%)	1.00
diabetes	12 (35.3%)	4 (23.5%)	8 (47.1%)	0.282
HbA1c ≥ 6.5%	10 (29.4%)	2 (11.8%)	8 (47.1%)	0.399
coronary artery disease	8 (23.5%)	5 (29.4%)	3 (17.6%)	0.688
other cardiac disease^b^	6 (17.6%)	5 (29.4%)	1 (5.9%)	0.175
chronic respiratory disease	5 (14.7%)	3 (17.6%)	2 (11.8%)	1.00
tobacco smoking	10 (29.4%)	6 (35.3%)	4 (23.5%)	0.708
chronic kidney failure	8 (23.5%)	6 (35.3%)	2 (11.8%)	0.225
cancer	8 (23.5%)	4 (23.5%)	4 (23.5%)	1.00
immunosuppression	4 (11.8%)	1 (5.9%)	3 (17.6%)	0.601
frailty^c^	7 (20.6%)	6 (35.3%)	1 (5.9%)	0.085

73.5% of the patients were privately insured with no difference between survivors and non-survivors. DNR order was given in five cases not excluding ventilation therapy for respiratory failure.

As expected, non-survivors exhibited a noticeable greater severity of disease depicted by Simplified Acute Physiology Score (SAPS) II score (50±7 vs. 42±14; p=0.037) or Sequential Organ Failure Assessment (SOFA) score (10.0±1.9 vs. 7.8±4.6; p=0.083).

Preeminently, the medical history of the patients comprised overweight (76.5%), hypertension (52.9%), diabetes (25.3%) and cardiac diseases (23.5% coronary artery disease, 17.6% valve operation or chronic heart failure). The number of patients with chronic respiratory precondition was low. Frailty occurred more often in the non-survivor group (35.3% vs. 5.9%; p=0.085). No significant differences were found between the two groups relating to the comorbidities.

Assessment of symptoms, vitals on admission, virologic findings, microbiological findings, the laboratory measures, and imaging are presented in Table 2.

**Table 2:** Clinical Workup Abbreviations: COVID-19, coronavirus disease 2019; CT, computed tomography; PCR, polymerase chain reaction; SARS-CoV-2, severe acute respiratory syndrome coronavirus 2. The clinical workup is presented for all patients. Data are n (%) or mean with standard deviation and range. Student´s t-test or Fisher´s exact test was performed to derive p-values. a Respiratory rate was assessed in case patient was not mechanically ventilated.

	all patients (n=34)	non-survivors (n=17)	survivors (n=17)	p-value
COVID-19 symptoms				
days from onset COVID-19 symptoms	8±3 (2-14)	8±4 (2-14)	8±3 (3-14)	0.75
contact to SARS-CoV-2 positive person	11 (32.4%)	8 (47.1%)	3 (17.6%)	0.071
traveled to a region with known transmission	4 (11.8%)	3 (17.6%)	1 (5.9%)	0.335
weakness	31 (91.2%)	15 (88.2%)	16 (94.1%)	1.00
shortness of breath	26 (76.5%)	11 (64.7%)	15 (88.2%)	0.172
reported fever	22 (64.7%)	10 (58.8%)	12 (70.6%)	0.682
cough	21 (61.8%)	10 (58.8%)	11 (64.7%)	0.704
diarrhea	8 (23.5%)	4 (23.5%)	4 (23.5%)	1.00
vitals on admission				
temperature (° Celsius)	37.7±1.0 (35.6-39.5)	37.5±1.2 (35.6-38.9)	37.9±0.9 (36.5-39.5)	0.231
heart rate (beats per minute)	89±22 (51-147)	90±24 (51-138)	87±21 (58-147)	0.729
mean arterial pressure (mmHg)	93±19 (59-135)	85±16 (67-127)	96±21 (59-135)	0.213
respiratory rate (breaths per minute)^a^	30±7 (15-44)	29±7 (15-36)	31±7 (22-44)	0.464
virologic findings (on admission)				
SARS-CoV-2 PCR positive	34 (100%)	17 (100%)	17 (100%)	1.00
SARS-CoV-2 swab positive	30 (88.2%)	16 (94.1%)	14 (82.4%)	0.601
SARS-CoV-2 tracheal secretion positive	9 (26.5%)	4 (23.5%)	5 (29.4%)	1.00
confirmed rule-out of viral co-infection	14 (41.2%)	6 (35.3%)	8 (47.1%)	0.486
microbiological findings				
initial blood culture negative	33 (97.1%)	1 (5.9%)	0	1.00
initial urine culture negative	25 (73.5%)	2 (11.8%)	1 (5.9%)	1.00
initial tracheal secretion physiological	20 (58.8%)	11 (64.7%)	9 (52.9%)	1.00
follow-up tracheal secretion physiological	8 (11.8%)	4 (23.5%)	4 (23.5%)	1.00
laboratory measures				
lymphopenia (<19%)	33 (97.1%)	17 (100%)	16 (94.1%)	1.00
lymphocytes (%)	8.0±5.0 (1.5-18.7)	7.6±4.2 (1.8-17.0)	8.6±5.3 (1.5-18.7)	0.545
white blood counts (Tsd/µl)	10.2±4.0 (4.0-23.0)	10.8±4.7 (6.0-23.0)	9.5±3.5 (4.0-16.0)	0.371
hemoglobin (g/dl)	12.4± (7.0-15.5)	12.5±1.7 (9.9-15.5)	12.4±2.6 (7.0-15.1)	0.834
HbA1c (%)	6.7±1.4 (4.9-11.9)	6.2±0.9 (4.9-8.3)	6.9±1.6 (5.6-11.9)	0.202
platelets (Tsd/µl)	222±96 (39-411)	202±94 (45-385)	241±97 (39-411)	0.247
international normalized ratio	1.26±0.56 (0.98-4.0)	1.39±0.76 (0.98-4.0)	1.14±0.19 (1.00-1.80)	0.190
D-Dimer (mg/l)	9.2±13.3 (0.5-49.0)	9.6±14.2 (0.85-49.0)	9.0±13.0 (0.5-45.0)	0.892
C-reactive protein (mg/dl)	147±13 (31-291)	147±78 (31-291)	146±70 (37-273)	0.972
procalcitonine (ng/ml)	0.60±0.85 (0.06-3.95)	0.71±0.9 (0.09-3.95)	0.52±0.81 (0.06-3.40)	0.531
interleucine 6 (pg/ml)	1034±3448 (22-20.149)	1602±4819 (52-20.149)	466±782 (22-2285)	0.344
creatinine (mg/dl)	1.50±0.98 (0.52-4.70)	1.61±1.12 (0.70-4.70)	1.46±0.86 (0.52-4.10)	0.665
lactate dehydrogenase (U/l)	464±173 (104-763)	520±192 (104-763)	408±135 (208-642)	0.056
aspartate aminotransferase (U/l)	93±18 (27-515)	82±44 (36-206)	103±142 (27-515)	0.557
alanine aminotransferase (U/l)	56±58 (9-283)	54±51 (16-234)	59±66 (9-283)	0.790
bilirubine (mg/dl)	0.9±0.9 (0.01-3.9)	0.9±0.8 (0.01-2.5)	1.0±1.0 (0.01-3.90)	0.674
pro brain natriuretic peptide (pg/ml)	1245±7153 (50-31.272)	6207±9272 (50- 31.272)	2869±3999 (50-14.270)	0.199
creatine kinase (U/l)	355±184 (24-4095)	529±1058 (45-4095)	171±218 (24-731)	0.191
troponin T (ng/l)	166±430 (5-2110)	201±532 (12-2110)	129±299 (5-1127)	0.658
arterial lactate (mmol/l)	1.6±1.5 (0.5-9.5)	1.4±0.6 (0.7-2.6)	1.9±2.0 (0.5-9.5)	0.364
imaging				
radiology				
chest radiography	32 (94.1%)	17 (100%)	15 (88%)	0.485
bilateral infiltration	32 (94.1%)	17 (100%)	15 (88%)	0.485
pleural effusion	5 (14.7%)	5 (29.4%)	0	0.048
congestion	3 (8.8%)	1 (5.9%)	2 (11.8%)	0.589
chest CT scan with bilateral ground-glass +/- consolidations	23 (67.6%)	12 (70.6%)	11 (64.7%)	1.00
contrasted chest CT scan	13 (38.2%)	6 (35.3%)	7 (42.2%)	1.00
ultrasound				
documented lung sonography with COVID-19 signs	28 (82.4%)	13 (76.5%)	15 (88.2%)	0.656
documented echocardiography	29 (85.3%)	15 (88.2%)	14 (82.4%)	1.00
unimpaired ejection fraction (EF ≥ 55%)	23 (67.6%)	11 (64.7%)	12 (70.6%)	1.00
impaired ejection fraction (EF < 55%)	6 (17.6%)	4 (23.5%)	2 (11.8%)	0.656

ICU resources and management

The reason for ICU admission was a moderate (50%) or severe ARDS (50%). Length of ICU treatment in non-survivors was 17±7 (2-72) days, patients discharged from ICU were treated 25±22 (1-81) days (Table 3).

**Table 3:** ICU Resources and Therapy Abbreviations: FiO2, fraction of inspired oxygen; ICU, intensive care unit; IMV, invasive mechanical ventilation; NIV, non-invasive ventilation; NIV +/- IMV or HFNC, high flow nasal cannula +/- IMV: Non-invasive strategy (NIV or HFNC) followed by intubation or as exclusive strategy during ICU treatment; PEEP, positive end-expiratory pressure; P insp, inspiratory pressure;  P peak, measured peak pressure; vv-ECMO, veno-venous extracorporeal membrane oxygenation (One patient received an arterio-venous ECMO because of persistent cardiogenic shock after preceding myocardial infarction). ICU resources and therapy are displayed for all patients, non-survivors and survivors. Data are n (%) or mean with standard deviation and range. Student´s t-test or Fisher´s exact test was performed to derive p-values. a Therapeutic positioning maneuvers included complete proning, incomplete proning (135°), pilot´s seat positioning or the use of a rotational bed. b Acute kidney injury is defined by the RIFLE definition [9].

	all patients (n=34)	non-survivors (n=17)	survivors (n=17)	p-value
length of ICU stay	21±19 (1-81)	17±17 (2-72)	25±22 (1-81)	0.275
ventilation				
NIV +/- IMV	13 (38.2%)	5 (29.4%)	8 (47.1%)	0.481
HFNC +/- IMV	13 (38.2%)	5 (29.4%)	8 (47.1%)	0.481
intubation	31 (91.2%)	17 (100%)	14 (82.4%)	0.227
IMV days	19±21 (0-79)	16±18 (1-77)	22±23 (0-79)	0.431
weaned respirator	14 (41.2%)	2 (11.8%)	12 (70.6%)	0.001
day 1 (at controlled ventilation with highest PEEP level)				
lowest PaO2/FiO2	114±33 (60-227)	109±42 (60-227)	129±19 (82-148)	0.346
FiO2 (%)	66±19 (40-100)	66±19 (40-100)	67±19 (40-100)	0.804
PEEP (mbar)	13±3 (7-18)	13±3 (7-18)	12±3 (7-17)	0.220
P peak (mbar)	28±5 (17-37)	29±5 (17-37)	27±5 (19-36)	0.345
compliance (ml/mbar)	51±28 (18-120)	51±30 (18-101)	50±28 (20-120)	0.992
resistance (mbar/sec)	12±4 (3-17)	12±3 (7-17)	12±4 (3-17)	0.647
tidal volume (ml)	460±130 (270-780)	490±140 (270-780)	430±120 (260-600)	0.239
first spontaneous breathing (> 10min.) in the first 24h hours	25 (73.5%)	10 (58.8%)	15 (88.2%)	0.118
vv-ECMO	8 (23.5%)	5 (29.4%)	3 (17.6%)	0.688
ECMO days	20±23 (2-75)	22±30 (2-75)	16±7 (8-20)	0.742
weaned ECMO	3 (8.8%)	0	3	0.018
therapeutic positioning				
overall therapeutic positioning maneuvers^a^	30 (88.2%)	16 (94.1%)	14 (82.4%)	0.601
proning	23 (67.6%)	12 (70.6%)	11 (64.7%)	1.00
rotational bed	2 (5.9%)	1 (5.9%)	1 (5.9%)	1.00
135°/pilot	5 (14.7%)	3 (17.6%)	2 (11.8%)	1.00
supine bedding	4 (11.8%)	1 (5.9%)	3 (17.6%)	0.601
acute kidney injury^b^	29 (85.3%)	15 (88.2%)	14 (82.4%)	1.00
renal replacement therapy	12 (35.3%)	6 (35.3%)	6 (35.3%)	1.00
overall vasopressor therapy	28 (82.4%)	17 (100%)	11 (64.7%)	0.018
no vasopressors	6 (17.6%)	0 (0%)	6 (35.3%)	0.018
low vasopressors	12 (35.3%)	4 (23.5%)	8 (47.1%)	0.282
mod. vasopressors	5 (14.7%)	4 (23.5%)	1 (5.9%)	0.335
high vasopressors	11 (32.4%)	9 (52.9%)	2 (11.8%)	0.026
packed red blood cells	17 (50%)	12 (70.6%)	5 (29.4%)	0.038

Attempts for non-invasive management (NIV or HFNC) before intubation were carried out in 38.2%.

Intubation was necessary in 91.2% of the cases. Three patients (8.8%) were exclusively managed with HFNC/NIV. On average, patients were on IMV for 19±21 (0-79) days. On day one of IMV therapy measured ventilation values showed a relatively unimpaired compliance 51±28 (18-120) (ml/mbar) and low resistance levels 12±4 (3-17) (mbar/sec).

The ventilation settings met lung protection on average (positive end-expiratory pressure (PEEP) 13±3 (7-18) mmHg, P peak 28±5 (17-37) mmHg, tidal volume 460±130 (270-780) ml), apart from relatively high fraction of inspired oxygen (FiO2) levels 66±19 (40-100) %. Spontaneous breathing on the ventilator was performed in 73.5% in the first 24 hours. Weaning from the respirator was successfully completed in 12 cases before ICU discharge, five patients went to a weaning unit.

Prone positioning was performed in 67.6%. If prone positioning was not indicated, alternative strategies were incomplete prone positioning (135°), pilot´s seat positioning or the use of a rotational bed. Thus, 88.2% of our patients underwent daily therapeutic positioning maneuvers.

If conservative therapeutic efforts failed, ECMO therapy was installed in 8/34 (23.5%) patients for 20±23 (2-75) days. Out of this subgroup, three patients were weaned from ECMO therapy. Two patients died of refractory ARDS and two because of intracerebral hemorrhage during ECMO, one patient died with abdominal sepsis and bleeding during ECMO therapy.

A noticeable number of patients showed an acute kidney injury (85.3%), whereas about 35.3% of all cases required RRT. Due to vasoplegic or cardiogenic shock, non-survivors significantly more often required vasopressor therapy (17/17 vs. 11/17; p=0.018) and transfusions of packed red blood cells (12/17 vs. 5/17; p=0.038).

Initially, antiviral treatment was applied to almost all patients (97.1%), but not continued later on, when it was proven not to be effective.

Resource intense multi-organ replacement ICU treatment was measured in nursing hours using Inpuls®-categories in 25 patients. A time investment of >20 hours per day was necessary in 96.0% of our patients and required a 1:1 nurse-patient ratio (Table 4).

**Table 4:** Nursing Hours Nursing hours per day (h/d) were assessed for 25 patients and are calculated according to Inpuls®, Intensivpflege- und Leistungserfassungssystem, intensive care nursing and activity recording system (Inpuls®, Heidelberg, Germany). Values were assessed at a representative day after admission. Data are displayed per category in n (%). Student´s t-test or Fisher´s exact test was performed to derive p-values.

category according to Inpuls® (nursing hours in h/d)	all patients with assessment (n=25)	non-survivors (n=12)	survivors (n=13)	p-value
1 (7.33 h/d)	0	0	0	
2 (8.40 h/d)	0	0	0	
3 (11.00 h/d)	1 (4.0%)	0	1	1.00
4 (13.85 h/d)	0	0	0	
5 (20.25 h/d)	14 (56.0%)	6 (35.3%)	8 (47.1%)	0.695
6 (21.66 h/d)	10 (40.0%)	6 (35.3%)	4 (23.5%)	0.428

**Figure 1:**
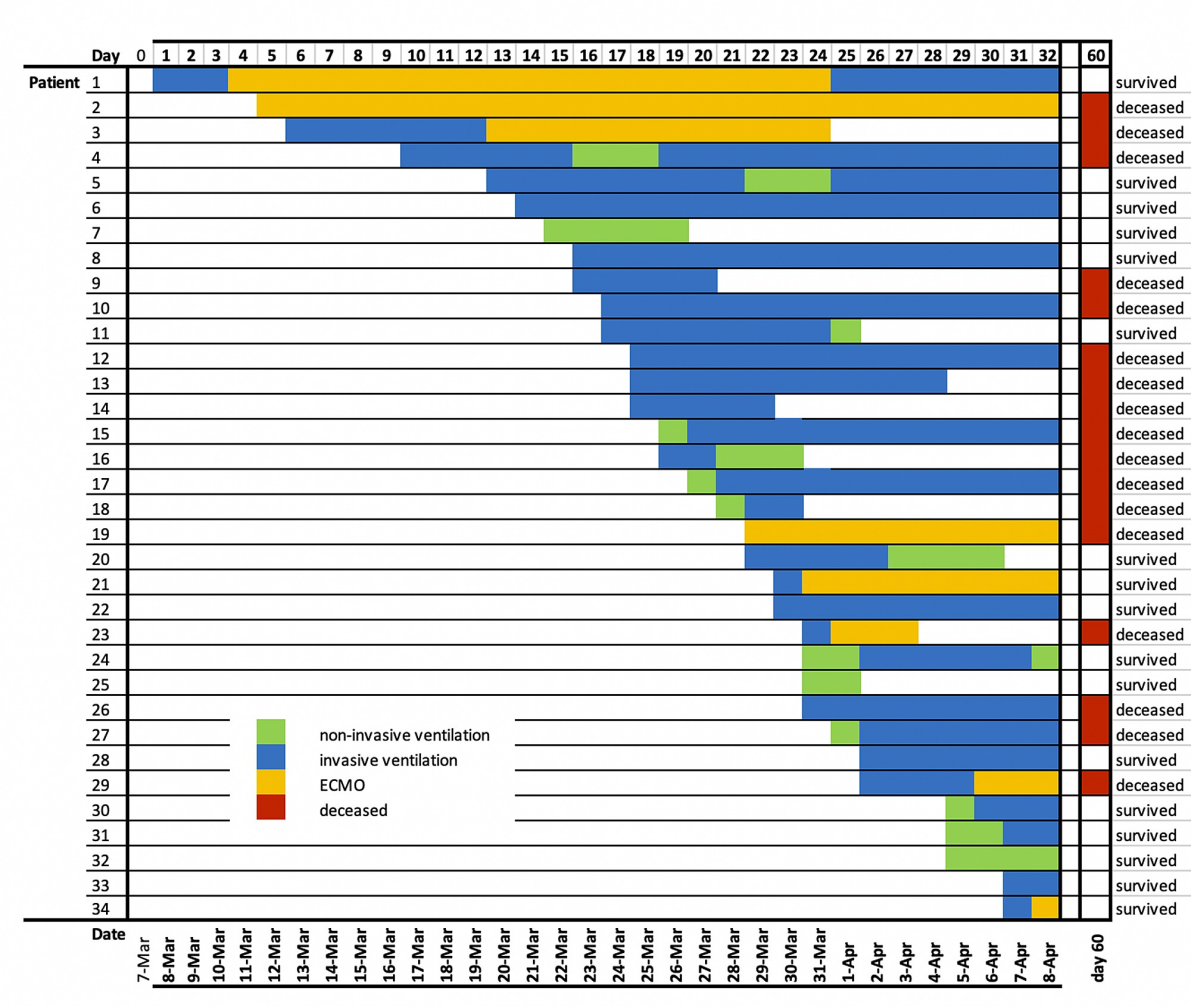
Clinical Courses and Outcomes at Day 60 of Critically-ill COVID-19 Patients Date of admission, clinical course and outcomes at day 60 are depicted for each patient from the first admission on 03/08/2020 until 05/28/2020. Days with non-invasive ventilation (non-invasive oxygen support; green), invasive ventilation (blue) and additional extracorporeal membrane oxygenation (ECMO; orange) are displayed.

To present the cumulative resources during the pandemic on the ICU, we depicted the dates of arrival, organ replacement therapies per patient and outcomes on day 60 (Figure 1).

**Figure 2:**
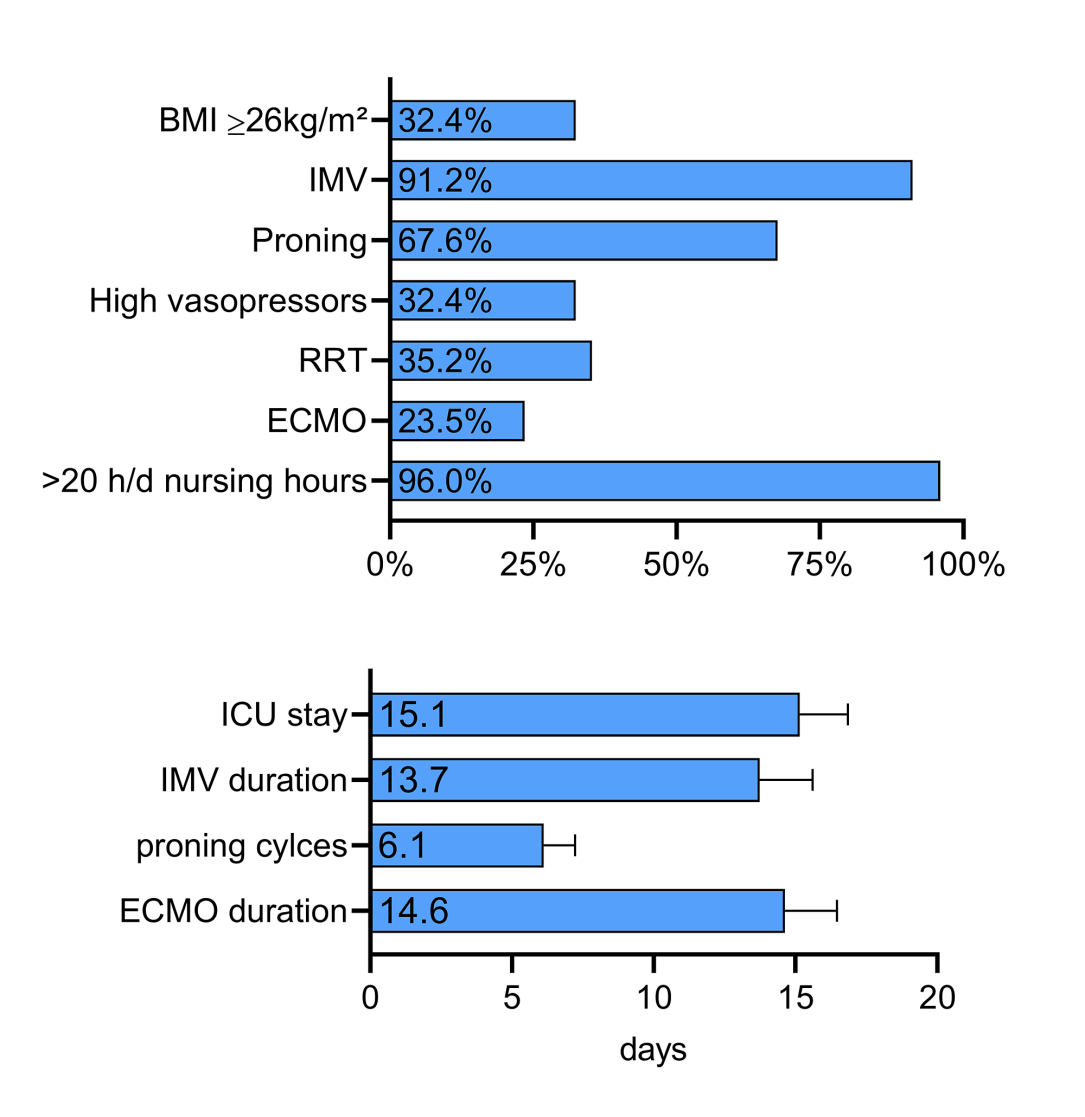
Intensive Care Resources and Therapies in Critically Ill COVID-19 Patients Cumulative intensive care resources and therapies are displayed in %. The average of length of stay, invasive mechanical ventilation (IMV) days, proning cycles, and days of extracorporeal membrane oxygenation (ECMO) therapy are displayed in days or cycles. RRT, renal replacement therapy.

The cumulative therapies of our units are displayed in Figure 2.

**Figure 3:**
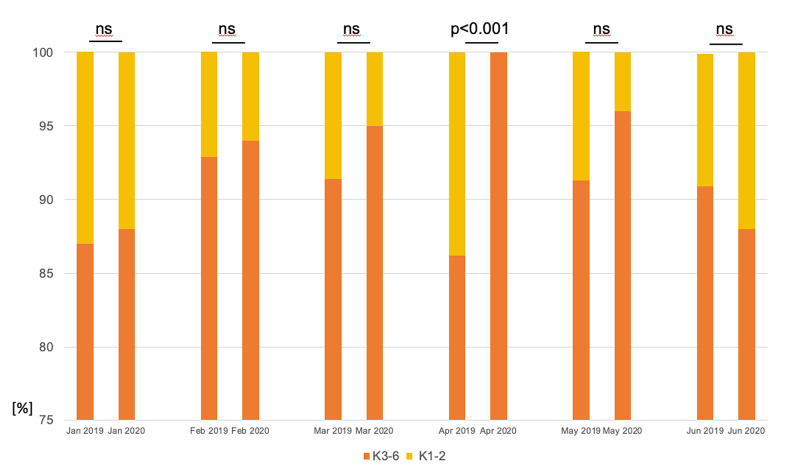
Nursing Burden in the First Wave of the COVID-19 Pandemic on a Medical ICU Inpuls® data show intensive care nursing and activity recording for an exemplary 14-bed medical ICU. Categories 1 and 2 (K1-2) include monitoring patients, categories 3-6 (K3-6) include intensive care patients with ascending maintenance effort, severity of disease, and resource application. Data is presented over the months January to June 2019 (baseline) and 2020 (first wave of the COVID-19 pandemic). In April the whole burden of the first wave of the COVID-19 pandemic is depicted as patients are categorized 100% in K3-6, whereas in 2019 representative values for this ICU are displayed with averagely 90% of the patients in K3-6.

Cumulative Inpuls® data show the nursing burden in the first wave of the COVID-19 pandemic for an exemplary medical ICU (14-beds). Data is presented over the months January to June 2019 (baseline) and 2020 (first wave of the COVID-19 pandemic) (Figure 3).

Whereas in 2019 representative values for this ICU are displayed with averagely 90% of the patients in K3-6 (intensive care patients with ascending maintenance effort, severity of disease, and resource application), in April 2020 patients are categorized exclusively (100%) in K3-6.

Outcomes and complications

At the end of the follow-up, exactly half of our patients were deceased, the other half was discharged from ICU, including five transferals to weaning units (four in-house transferals). More than half of the survivors were able to be discharged home (58.8%), two went on a rehabilitation therapy without oxygen supply. On average the followed-up survival since admission was 60±8 days (51-81 days).

The clinical course was complicated by pneumothorax or pneumomediastinum in six (17.6%) cases. Furthermore, five (14.7%) patients exhibited non-fatal pulmonary embolism/thrombosis in segmental and sub-segmental lung arteries. Superinfections during treatment in 18 (52.9%) cases were dominated by *Serratia marcescens* (33.3%). *Aspergillus fumigatus* superinfection was detected in two (11.1%) patients (Table 6).

**Table 5:** Complications and Outcomes Complications and outcomes are presented as n (%) or mean with standard deviation and range. Fisher´s exact test was performed to derive p-values. a Other superinfections included Haemophilus influenza, Streptokokkus pneumonia, Staphylococcus aureus, Pseudomonas aeruginosa. b Cause of death was analyzed for the 17 deceased patients according to clinical judgement. No medical autopsies were carried out. Data are n (%). DNR order, do not resuscitate order. c Transferals to weaning units included four transferals to an incorporated weaning unit and one transferal to an external weaning unit.

	all patients (n=34)	non-survivor (n=17)	ICU-survivors (n=17)	p-value
complications				
respiratory superinfection (initially)	11 (32.4%)	6 (35.3%)	5 (29.4%)	1.00
respiratory superinfection (during treatment)	18 (52.9%)	9 (52.9%)	9 (52.9%)	1.00
Serratia marcescens	6 (17.6%)	3 (17.6%)	3 (17.6%)	1.00
Aspergillus fumigatus	2 (5.9%)	2 (11.8%)	0 (0%)	0.485
other superinfection^a^	10 (29.4%)	4 (23.5%)	6 (35.3%)	0.708
pneumothoraxes/pneumomediastinum	6 (17.6%)	4 (23.5%)	2 (11.8%)	0.656
pulmonary embolism	5 (14.7%)	2 (11.8%)	3 (17.6%)	0.608
intracerebral hemorrhage	5 (14.7%)	5 (29.4%)	0 (0%)	0.044
delirium	4 (11.8%)	1 (5.9%)	3 (17.6%)	0.601
cause of death^b^				
refractory respiratory failure		3 (17.6%)		
refractory multiorgan failure		9 (52.9%)		
fatal intracerebral hemorrhage		3 (17.6%)		
withdrawal due to DNR order		2 (11.8%)		
discharge place				
weaning unit^c^			5 (29.4%)	
rehabilitation (without weaning)			2 (17.6%)	
home			10 (58.8%)	
survival since admission, days			60±8 (51-81)	

Intracerebral hemorrhage was significantly more often recorded in non-survivors (p=0.044).

Nine (52.9%) patients died because of untreatable multi-organ failure as the main cause of death in the cohort.

Three (21.4%) patients died because of refractory ARDS. Notably, 3/17 (17.6%) patients died of fatal intracerebral hemorrhage. Because of SARS-CoV-2 PCR positivity, organ donation was impossible in these patients. Two (11.8%) patients did not continue therapy, as they were not willing to undergo re-intubation and received palliative treatment. No medical autopsies were carried out in this cohort.

## Discussion

Intensive care resources and 60-day survival

We present a 60-day follow-up of our first critically ill COVID-19 patients due to pulmonary failure in southwestern Germany. The age and common comorbidities were similar to international cohorts [1-3,12]. In our cohort, 60-day survival was 50.0%. Importantly, the majority of our survivors were able to return to their former lives.

This is similar to the case-fatality rate of 49.0% reported for “critical cases” in a large Wuhan registry [13] and much lower than suggested by early reports in the COVID-19 pandemic for patients with severe pulmonary failure [2,3]. Importantly, we present data with a long follow-up. In other registries a very small number of patients actually could return to normal life [2,3].

To characterize the non-survivor and possible determinants of mortality at 60 days, we analyzed the mode of death. Data on mode of death for in-hospital mortality were presented beforehand [5]. Thus, respiratory failure remained eminent in three cases. All other patients seemed to have overcome respiratory failure but died from overwhelming complications or multiorgan-failure. Interestingly, three patients died from intracerebral hemorrhage and were studied in a subsequent analysis [14]. ICU therapy withdrawal was due to DNR orders in two patients (no re-intubation preferred) - not decisively influencing the high mortality in our opinion. Patients not willing to receive ICU therapy were primarily not transferred to our unit. If patients developed the wish to not continue ICU therapy, we adhered to their wish. 

Data of 92 COVID-19-associated deaths states ARDS as the main complication followed by myocardial injury, liver injury, kidney injury, and multiple organ failure [15]. In pandemic conditions, resource shortages might necessitate rapid decisions regarding futility compared to normal conditions. Survival rate in the present study is lower than described for a general ARDS population (60-day survival 68%) [16]. When considering that ARDS mortality is lower in tertiary hospitals than in hospitals of first and second treatment level [17], the high mortality found in this registry might suggest a significant case-fatality rate of COVID-19 patients requiring ICU therapy. According to the severity scores (SOFA and SAPS II score), our cohort had a relatively high predicted risk of death (over 40%), which is higher than in cohorts characterized beforehand.

Finally, we do not know if unlimited resource allocation affects mortality. We can only suggest that reported very high death rates in the first weeks of the pandemic might result from a shortage of resources and a collapsing health system.

But, no matter what kind of resources you apply, death rates seem to stay about 50% in a very critical ICU cohort. This further depicts the potential harm of the virus and possible devastating course of COVID-19.

Other general determinants of mortality are age, comorbidities, multi-organ failure, and complications. The latter are often due to a hypercoagulable state during the infection. As stated by Klok et al. the incidence of thrombotic complications is up to 31% in COVID-19 patients on the ICU [4]. The notable rate of 14.7% non-fatal pulmonary artery thrombosis is in line with their findings. Further studies concentrated on autopsies and confirmed a high rate of thromboembolic events [18]. At the moment, the underlying mechanism for the coagulopathy leading to pulmonary artery thrombosis and cerebral hemorrhage, which were leading causes of the complications in this cohort, is unknown.

As Germany did not experience a shortage of ICU resources, it is not surprising that in our cohort ICU therapy lasted longer than reported as in other registries [1,19]. Not knowing the course of the disease, we ran through long therapies with our patients - partly being surprised by their recovery capacities after weeklong efforts and stagnation.

Also, more resources were allocated to patients in this registry including multiple organ replacement therapies - ECMO therapies and RRT were required more often than reported in other cohorts from Wuhan or New York [1,2]. Another German registry depicts similar rates of ECMO and RRT [3].

Our hospital never faced a personal or machine shortage (including ventilators, dialysis machines or ECMO systems). Staffing acquirements were calculated by the Inpuls® category and met with 2:1 (patient:nurse) or in extreme cases 1:1 (patient:nurse) staffing. We are the first to report an estimate on the enormous amounts of nursing hours required for treating every patient and the burden for a whole ICU unit during the first wave of the COVID-19 pandemic.

If resource allocation was appropriate remains unknown. The long courses of disease and late possible recoveries suggest not to limit ICU therapies to a certain time span or compare to other virus or bacterial pneumonia. 

This might be even more difficult if facing a resource shortage. Concepts of resource allocation should be discussed interdisciplinary according to current recommendations and local conditions/obligations [20].

Limitations

The case number is limited and the data has to be verified by larger cohorts and longer follow-up intervals. Further, the cohort is a selected one as the university hospital of Freiburg is a tertiary center. Therefore, generalizability for other settings is limited. The center has a large number of patient allocations from primary or secondary treatment centers because of evaluation of ECMO therapy or limited resources in the other centers. For comparability patients´ severity scores should be regarded e.g., SOFA Score.

Moreover, our study misses further information about patients´ socioeconomic status. To hint at income level, we included the health insurance status. In Germany private health insurance is only allowed after reaching a certain yearly income level (e.g., 62.550 Euro in 2020).

Also, as many patients were transferred from other hospitals a loss of information is not excludable in therapies prior to ICU.

Finally, 60-day mortality was incomplete followed if patients were discharged home earlier. Further, follow-up was not assessed by functional state and questionnaires on quality of life.

## Conclusions

Caring for critically ill COVID-19 patients requires an immense amount of organ replacement therapies, nursing hours and ICU days. The 60-day survival is 50% in a German tertiary treatment center despite full resources. High mortality in COVID-19 might be disease-specific rather than caused by resource shortage.
